# Hypodermic Medication

**Published:** 1875-01

**Authors:** J. B. Mattison

**Affiliations:** Chester, N.J.


Vol. V.—JANUARY, 1875.—No. 1.

### Original Communications.

### HYPODERMIC MEDICATION.

BY J. B. MATTISON, M. D., OF CHESTER, N. J.

In the September number of the New York *Medical Journal* is a captiously critical article, by Dr. Stephen Rogers, on the above subject, having *a special* reference, however, to *quinine* solutions, so administered, but in which the writer, towards the close, abandoning the main line of assault, goes off in, what we consider, a most unwarrantable attack on subcutaneous medication in general.

The principal target for his professional firing seems to have been an instructive article by Dr. Leute, in the March number of the same journal, on "Hypodermic Injections of Quinine in Intermittent Fever," and which appears to have convinced Dr. R. that danger still confronts the profession, and, on account thereof, he deems it his bounden duty to raise the voice of protest and appeal.

Condemnatory of quinine hypodermically, Dr. Rogers claims to sermonize from no new text. For a quarter score of years he has at times borne testimony to the pernicious effects of such medication, and, from a paragraph in his recent communication, "the rusty and neglected hypodermic syringes formerly so actively employed by the expert hands of our professional acquaintances," we are warranted in inferring that his good offices in this direction have not fallen on the hither side of the object intended.

It is not the purport of this article to combat the assertions of this reviewer as regards the subcutaneous use of quinine, be the solvent what it may, although it would be far from difficult to quote numerous authorities who have given the most emphatic testimony in its favor. We desire, the rather, to enter an opinion strongly at variance with the closing statements of Dr. Rogers' paper, in the which he says: "Few of the fashions, however, from the days of Sangrado's bleeding and hot water, have been carried to more ridiculous and destructive extremes, than this wild fashion of medicating mankind under the skin. Fatal results, strictly attributable to this method, have frequently occurred from the commencement of the fashion, and bare escapes from this calamity in still greater numbers. These consequences, not to allude to abcesses, obstinate ulcers, and chronic and disabling indurations, have induced misguided but prudent physicians in great numbers to abandon this mode and lay aside their syringes. They see that disease can be treated quite as successfully, and at less risk of doing irreparable harm, without it, though they have occasion to deplore the loss of the *eclat* which the flourish of instruments and the production of pain were wont to bring them. While this is true of many, unfortunately for humanity there are still too many practitioners who are pursuing this method with all the ardor of a new idea."

Now, with no lack of respect for the author of the above quotation, we most certainly think he is putting his case too strongly, and beg leave to dissent *very thoroughly* from the opinion advanced and maintained. It is entirely *too sweeping*, and we question whether, in the full light of clinical experience, it would bear the scrutiny of careful investigation.

It is not ours to deny that unpleasant and unfortunate results have been charged—not unjustly, it may be—to the use of the hypodermic syringe; but does it follow therefrom that the *whole* system of hypodermic medication is to be denounced and discarded? Must the subcutaneous administration of medicines, in its *entirety*, be abrogated because some over-zealous disciples of Wood see fit to carry their pet project to an unwarrantable extreme? By no means. As well expunge chloroform or ether from the Pharmacopœia, for it is an undeniable fact that deaths by the dozen have resulted from the administration of these invaluable agents, and that too, at times, in the hands of professional men careful and competent. We once saw, in the amphitheatre of Bellevue Hospital, a hale and hearty Irishwoman, on whom the Talicotian operation had been performed by Professor Frank H. Hamilton, gasp and die almost immediately, despite the most strenuous averting efforts, on the administration of chloroform, which was resorted to after a previous trial of ether, to which the patient was found unyielding—this, too, under the supervision of a surgeon proverbially careful in his administration of anæsthetics, and whose numerous classes can bear abundant testimony to the stress he was wont to lay on the cautious giving of these powerful pain preventives. But was this unfortunate result the means of placing chloroform on the index expurgatories of that institution? Most assuredly not. Carry out such a law to its ultimatum, and we would speedily find our stock in trade reduced to its minimum.

Attributable to the hypodermic method, Dr. Rogers says: "These consequences (fatal results and bare escapes), not to allude to abscesses, obstinate ulcers, and chronic and disabling indurations, have induced misguided but prudent physicians in great numbers to abandon this mode." It must be admitted that this is rather a formidable array of objectionable sequelæ; but is the testimony of the profession at large *pro* or *con* on this point? Speaking individually, we must answer *most emphatically* in the *negative*, and our experience has not been a very limited one. For years we have employed the hypodermic syringe, and so highly do we prize it that we deem it one of the most valuable weapons in our professional armamentarium, and do not consider ourself "armed and equipped as the law directs" without it.

Notwithstanding all this, we distinctly disclaim that degree of "infatuation which makes it positively hazardous for a person with a pain or an ache to be near us." It not at all follows that our exalted opinion of this mode of medication makes us proceed to puncture every ailing one about us, as if we were a sort of perambulating apiary.

The sum total of resultant "casualties" has been two small, circumscribed abscesses of the forearm, and these occurred during the past two months in the same patient, a male of unusually fine *physique*, and in whom they occasioned no more discomfort than an ordinary phlegmon, and not at all comparable to the torture (hepatic colic) which a single injection of morphia, large it is true, one grain, speedily sufficed to relieve.

We have cognizance of another case, somewhat notorious, in which the subject had succumbed to an opium habit, morphia hypodermically, and who, during nearly seven years' servitude, gave himself several thousand injections, with nothing more resulting locally than a number of limited inflammations, none of which proved formidable.

Eulenberg, with an experience of many thousand cases, noticed *three times only* inflammation of the punctured spot, all due to the irritant nature of the fluid injected.

We will admit that our fortunate exemption may have been due to the non-irritating qualities of the liquids inserted; and yet, in the two cases mentioned, the injected material was simply an aqueous solution of morphia and atropia.

The medicinal agents we have employed are morphia, atropia, strychnia, quinia and chloroform. As a solvent for the first two, water; with the third, the same, adding a sufficient quantity of acetic acid to make a clear solution; with the quinine, acid sulph. dil., quantity sufficient to form a neutral solution, *freshly* made; and the chloroform was employed pure. Of all these, the solutions first named would seem to be the ones least likely to irritate; and yet, quite to the contrary, they set up some inflammation. Not the slightest ill effect followed the quinia or strychnine, and the chloroform, while it acted anæsthetically on the part injected, gave rise to nothing like an abscess. Less frequent than the others, by far, has been the application of the last mentioned remedy in the field of subcutaneous therapeutics. Indeed, foreign observers having experimented, condemned it for hypodermic purposes, deeming it ineligible on account of its actively irritant local effects, it remained for Prof. Bartholow to bring it forward a year ago with astonishing success in the treatment of tic douloureux. Three cases, in which this disorder existed in an aggravated degree, were completely cured; and his statements were verified, and the result fully confirmed, by the report of a case which we gave in the New York *Medical Record*, May 1, 1874, in which a single injection was sufficient to control a most atrocious attack of this agonizing disease. These four cases are the only ones on record, to our knowledge, of chloroform thus employed. It remains to be seen whether the same degree of success will attend its further use, or if, from an inappropriate application, it will fail of yielding results as eminently satisfactory as have attended its employment in trifacial neuralgia.

Dr. Rogers further says, speaking of those "misguided but prudent" practitioners who have abandoned hypodermic medication: "They see that disease can be treated quite as successfully without it." To this we must again withhold assent. It may be the experience of those to whom Dr. R. alludes, but we do *not* think it such of the great *body* of the profession. Speaking personally, we *know* we have accomplished *with* the hypodermic syringe what we *never* did *without* it, and what we think we never shall until some mode of medication superior even to this shall be presented to the fraternity.

Take, for instance, the case of biliary calculi to which we have referred. Several times that patient has been in our hands for treatment, and we have demonstrated to our *entire* satisfaction that the opiate treatment, which is accredited the most effectual *per orem*, will *not* bring to this man the relief obtainable by the subcutaneous injection of morphine. Nor is this an exceptional example. Cases of neuralgia, myalgia, sporadic cholera and intestinal colic have given way more speedily to hypodermic medication than any other form of treatment we ever employed. In the first mentioned disease, we have seen cases—one of long standing—of the facial variety, cured in five minutes by a single injection of morphia, followed by a blister to the nucha. We say cured, in the full sense of the term—the pain departing and failing to return. We challenge a similar result from any form of opium, time and amount the same, *per via nat.*

Take the case in which the injection of chloroform was followed by such a remarkable effect. Is it at all probable that its administration in any other manner would have achieved a like success? We think not.

Again, it is an indisputable fact that there are those in whom, owing to some idiosyncrasy, opium in any form—the comp. tinct. sometimes excepted—cannot be tolerated, and here the injection of morphia subcutaneously, in urgent cases, becomes a boon unobtainable in any other way. One notable example of this kind has fallen under our observation.

We repeat, then, our disbelief that diseases in which the hypodermic injection of morphia, or any of those other subcutaneous remedies which clinical experience has proven eligible, is not contraindicated, can be treated as successfully without as with this method. Ultimately, in many instances, the same result may be reached, but by no means as speedily, and we hazard little in opining that the *eclat* which Dr. Rogers says those who have abandoned the hypodermic syringe mourn the loss, is *not* due to the "flourish of instruments and production of pain," but to *speedy relief* from bodily anguish and distress. We commend to his consideration an able, exhaustive and instructive article on the subject which captions this communication, by Dr. J. C. Bishop, of Ohio, in the June number of this journal, and the report of the Standing Committee in the last Transactions of the New Jersey State Medical Society.

From what we have written, it will readily be concluded that we are a follower of the "wild fashion," which we make no haste to disavow that we are pursuing this method, *not* "unfortunately for humanity," but knowing full well that by its means we have been instrumental in ministering abundantly and acceptably to the relief of some of the many ills to which human flesh is heir.

---

"MARASMUS" OF CHILDREN.—*Pancreatic Emulsion.*—In those wretched cases in children, in which every part wastes except the abdomen, pancreatic emulsion in doses of a teaspoonful every four hours is often of signal benefit. It is, of course, necessary that a proper diet should be insisted on at the same time, but proper diet without the pancreatic emulsion will not do.—*Dr. Dobell.*

